# Thyroid Nodules and Obesity

**DOI:** 10.3390/life13061292

**Published:** 2023-05-31

**Authors:** Elpida Demetriou, Maria Fokou, Savvas Frangos, Panagiotis Papageorgis, Panayiotis A. Economides, Aliki Economides

**Affiliations:** 1 Department of Medicine, School of Medicine, European University Cyprus, 2404 Nicosia, Cyprus; ed181861@students.euc.ac.cy (E.D.); mf162105@students.euc.ac.cy (M.F.); p.economides@euc.ac.cy (P.A.E.); 2 Nuclear Medicine Department and Thyroid Cancer Clinic, Bank of Cyprus Oncology Center, 2404 Nicosia, Cyprus; savvas.frangos@bococ.org.cy; 3 Department of Life Sciences, European University Cyprus, 2404 Nicosia, Cyprus; p.papageorgis@euc.ac.cy; 4 Economides Thyroid and Endocrinology Center, Engomi, 2404 Nicosia, Cyprus; 5 Department of Health Sciences, European University Cyprus, 2404 Nicosia, Cyprus

**Keywords:** thyroid nodules, obesity, inflammation, leptin, estrogen, cytokines, metabolic syndrome, carcinogenesis

## Abstract

A widely discussed topic in the pathophysiology of thyroid nodules is the role of obesity, a state that leads to increased systemic inflammatory markers. Leptin plays a vital role in forming thyroid nodules and cancer through several mechanisms. Together with chronic inflammation, there is an augmentation in the secretion of tumor necrosis factor (TNF) and the cytokine interleukin 6 (IL-6), which contributed to cancer development, progression and metastasis. In addition, leptin exerts a modulatory action in the growth, proliferation and invasion of thyroid carcinoma cell lines via activating various signal pathways, such as Janus kinase/signal transducer and activator of transcription, mitogen-activated protein kinase (MAPK) and/or phosphoinositide 3-kinase (PI3K)/protein kinase B (Akt). Through several proposed mechanisms, aberrant endogenous estrogen levels have been suggested to play a vital role in the development of both benign and malignant nodules. Metabolic syndrome triggers the development of thyroid nodules by stimulating thyroid proliferation and angiogenesis due to hyperinsulinemia, hyperglycemia and dyslipidemia. Insulin resistance influences the distribution and structure of the thyroid blood vessels. Insulin growth factor 1 (IGF-1) and insulin affect the regulation of the expression of thyroid genes and the proliferation and differentiation of thyroid cells. TSH can promote the differentiation of pre-adipocytes to mature adipocytes but also, in the presence of insulin, TSH possesses mitogenic properties. This review aims to summarize the underlying mechanisms explaining the role of obesity in the pathophysiology of thyroid nodules and discuss potential clinical implications.

## 1. Introduction

Thyroid nodule prevalence has been rising along with a parallel increase in obesity worldwide over the past few decades [1,2,3,4]. Together with the rising incidence of thyroid nodules, thyroid cancer detection has rapidly increased [5]. The low-grade, chronic inflammation commonly observed during obesity leads to a non-specific immune response by increasing systemic inflammatory molecules, which contributes to developing thyroid nodules and cancer [6,7]. The increased release of leptin and decreased secretion of adiponectin from the white adipose tissue of obese individuals, along with high oxidative stress, enhances cell proliferation and angiogenesis [8]. Aberrant endogenous estrogen levels have been suggested to play a vital role in the development of both benign and malignant nodules. Obesity leads to increased aromatase activity in the peripheral adipose tissue resulting in higher levels of circulating estrogens. Several pathways are involved in the molecular effects of estrogen in follicular cells. Estrogens have a regulatory effect on vascular endothelial growth factor (VEGF) secretion in thyroid cells that increases angiogenesis [9]. Metabolic syndrome stimulates thyroid proliferation and angiogenesis due to hyperinsulinemia, hyperglycemia and dyslipidemia [10,11]. It triggers the secretion of IGF-1, and insulin regulates the expression of thyroid genes to control the proliferation and differentiation of thyroid cells. Furthermore, expression of TSH receptors in extrathyroidal tissues (specifically in adipose tissue) means that TSH can promote the differentiation of pre-adipocytes to mature adipocytes, and in the presence of insulin, TSH exerts mitogenic properties [12]. In this review, we summarize the latest evidence regarding the molecular links between obesity and thyroid cancer development and discuss emerging diagnostic and therapeutic implications.

## 2. Epidemiological Evidence Linking Thyroid Nodules, Thyroid Cancer and Obesity

Relevant information was searched for in Google Scholar and PubMed using the terms “obesity”, “adiposity”, “BMI,” and “thyroid nodules”. Our literature search did not impose any specific time frame for the included studies and we did not apply specific exclusion criteria to include all available studies relevant to the topic.

Many studies have demonstrated a link between obesity and thyroid nodules, especially in women. Some studies though, have failed to establish this association. In a community-based Chinese population study, waist circumference was associated with an increased thyroid nodule risk and waist circumference had a stronger association than BMI [13]. A cross-sectional observational study in Italy indicated an association between obesity, diabetes and thyroid nodules in females and older individuals. Obese people had a larger dominant nodule than nonobese participants [11]. Panagiotou G. et al. highlighted a higher frequency of nodular thyroid disease in overweight and obese individuals than those with normal weight. Age and female gender were independent predictors of nodular thyroid disease, along with specific sonographic features such as hypoechogenicity and vascularity [14]. In a large study involving 9203 subjects consisting of 6793 adults and 2410 children, a positive association between thyroid nodule risk and weight, height, BMI, and body surface area (BSA) was seen mainly among female subjects; the same effect was seen in children as well. BSA exhibited a more robust association, suggesting that taller and obese individuals are more vulnerable to developing thyroid nodules [15]. A cross-sectional study involving lean children indicated that higher levels of abdominal adiposity, as measured by BMI, body surface area (BSA), and waist circumference (WC), were significantly associated with an increased risk of thyroid nodules, particularly multiple nodules in girls and solitary nodules in boys [16]. In young individuals aged 21 years or younger, a higher BMI was associated with a higher risk of malignant neoplasms in thyroid nodules. The authors suggested that pediatric thyroid cancer follows a distinct disease process compared with its adult counterpart [17].

The association between obesity and thyroid nodules was not seen in a retrospective study, and they even observed that there is a possibility that severe obesity may have a protective effect against thyroid cancer. The study evaluated the results of fine-needle aspiration cytology (FNAC) from thyroid nodules in 4849 patients [18]. Layegh P. et al. also found no association between thyroid nodules in obese and nonobese individuals; although, among the study participants, those with insulin resistance had a higher prevalence of thyroid nodules. In the same study, thyroid volume was primarily associated with BMI [19]. The same was seen in a study focusing on morbidly obese individuals, indicating that women with morbid obesity have a notably lower prevalence and fewer solid thyroid nodules [20].

To better understand the correlation between obesity and thyroid nodules, we also reviewed studies that aimed to investigate fluctuations in BMI and other parameters of metabolic syndrome in correlation with their impact on the thyroid gland. In a large-scale study with 67,781 participants in China, subjects with worse metabolic profiles had more and larger thyroid nodules than those with a single nodule or nodules smaller than 1 cm. The metabolic components included BMI, systolic blood pressure (SBP), diastolic blood pressure (SBP), triglycerides (TG), high-density lipoprotein (HDL), low-density lipoprotein (LDL) and fasting glucose [21]. The results of another recent large-scale study by Lai et al. revealed that with every 5% increase in body fat percentage (BFP), 5 cm increase in height, 10 kg increase in weight and 0.05 m^2^ increase in body surface area (BSA) there is an increased risk of thyroid nodules both in males and females. At the same time, the same applies for every 5 Kg/m^2^ of BMI in men [22]. New evidence supports that the level of BMI influences the dimensions of thyroid nodules, since a higher BMI was associated with greater mean nodule dimensions [23].

BMI has a positive correlation not only with thyroid nodules but also with the risk of developing thyroid cancer [24]. BMI is a significant predictor for PTC in both men and women since those overweight and obese had a significantly higher risk of having a malignant nodule than those with normal weight. Moreover, advanced TNM stage was more common in higher BMI groups in the same study. At the same time, multifocality was the least common and nodule diameter was the smallest in those with normal weight. Hence, BMI is assumed to impact PTC tumors’ number, size and aggressiveness [25].

In a study conducted by Liu et al., a correlation between metabolic syndrome and thyroid nodules was found. The theory that the more components of metabolic syndrome an individual has, the greater the risk of thyroid nodules, was confirmed for patients with two or more criteria of metabolic syndrome. Moreover, in the same study, in the group with waist circumference (WC) ≥90 cm, a significant rise was observed in the incidence of thyroid nodules and the levels of TSH [26]. A new prospective study aiming to investigate the association of metabolic syndrome and thyroid nodules in individuals who recovered from metabolic syndrome revealed that this group had a similar risk as those free of the syndrome [27]. Furthermore, a prospective study with a follow-up of 6 months revealed improvement in thyroid parameters after obesity treatment. A decrease in both TSH and thyroid volume was observed in those with ≥10% weight loss, whereas in those with less weight loss, the results for these parameters were not statistically significant [28]. Considering the results of these studies, the impact of obesity and obesity-related parameters is profound. Both the observed progression of thyroid disease and its reversion with changes in obesity status highlight the importance of understanding the pathophysiology of the disease. With this information, new advancements in disease treatment strategies could be achieved.

Thyroid nodularity is common, with an increased prevalence worldwide. Palpable thyroid nodules are present in 3–7% of the general population [29]. The prevalence of thyroid nodules by high-resolution ultrasound is 19–68% in randomly selected individuals, with annually rising trends globally [30]. As expected, the detection rates of thyroid nodules are higher with ultrasound when compared to palpation since there has been a widespread application of sensitive imaging techniques that diagnose thyroid nodules in recent years [30,31]. Thyroid nodules are widespread, found in around 50–65% of healthy individuals, most often without symptoms. Most of these nodules are benign, with less than 7−15% of them being malignant [32,33,34]. The rise is mainly attributed to the widespread use of high-resolution ultrasonography and fine-needle aspiration biopsy, particularly for smaller tumors. However, it is important to note that the behavior and aggressiveness of thyroid tumors can vary, even among microcarcinomas [32]. In a study conducted by our group, an unexpectedly high prevalence of aggressive features was observed in multifocal “small” PTMCs of less than 5 mm. [35].

Several studies have shown that the prevalence of thyroid nodules depends on risk factors such as age, gender, iodine intake and history of radiation exposure [36]. In the United States, it is estimated that approximately half of adults older than 60 years have thyroid nodules. Although there is a correlation between advanced age and a high prevalence rate of thyroid nodules, the etiology of this relationship is unclear [37]. A relatively recent multicenter study in Korea (N = 72,319) found that the prevalence of thyroid nodules detected by ultrasound at health check-ups was 34%, rising to 55% for patients aged ≥70 years [31]. Female gender has also been proven to be a strong risk factor for thyroid nodules. The gender differences in thyroid nodules might be due to physiology, pregnancy and female estrogen exposure [38]. It is worth noting that the development of thyroid nodules is four times more frequent in women than men and that their prevalence increases with age and body mass index [39]. Another risk factor that influences the presence of thyroid nodules is iodine intake, which has been the subject of numerous studies. Thyroid nodules were found to be more prevalent in iodine-deficient countries. It has been reported that the prevalence of thyroid nodules ranged from 2.6% in iodine-sufficient countries to 20.2% in iodine-deficient areas [40]. In addition, it has been observed that hypertension, elevated fasting glucose levels and diabetes are associated with an increased risk of developing thyroid nodules. On the contrary, certain factors such as smoking, alcohol consumption, regular exercise, and following a Mediterranean diet appear to have potential protective effects against thyroid nodules [21,41].

Thyroid cancer represents the most common endocrine malignancy, with rising incidence and mortality globally. Thyroid cancer is the ninth most common type in men and the fifth most common cancer among women [42]. According to data from the International Agency for Research on Cancer (IARC), the global incidence of thyroid cancer was approximately 6.7 cases per 100,000 people in 2018 [43]. Approximately 90% of all thyroid malignancies are differentiated thyroid carcinoma, comprising papillary thyroid carcinoma (PTC) and follicular thyroid carcinoma (FTC). Poorly differentiated thyroid carcinoma (PDTC) and anaplastic thyroid carcinoma (ATC) are rare tumors accounting for 5% and 1%, respectively. Medullary thyroid carcinoma (MTC) represents 5% of thyroid cancers [44]. The incidence rates of FTC, ATC and MTC thyroid carcinomas have remained relatively stable over the past 30 years [45]. PTC has the highest rate of growth among all types of malignancies. Thyroid cancer has a generally favorable prognosis with a 5-year papillary thyroid carcinoma survival rate of over 95% [46]. In the United States, the incidence of thyroid cancer has tripled over the last thirty years, with an annual growth rate of approximately 3% from 1974 to 2013. According to the American Cancer Society’s 2023 estimates, there were about 43,720 new cases of thyroid cancer, 12,540 in men and 31,180 in women. Almost 1 million people (915,664) had thyroid cancer in 2019; approximately 2230 deaths from thyroid cancer were recorded in the United States in 2022. New thyroid cancer diagnoses increased steadily between the 1990s and 2010, when the average number of new cases decreased. There was a rise in death rates between 2009 and 2018 but they have stayed low. The Global Cancer Observatory estimated there were 586,000 new cases of thyroid cancer worldwide in 2020. The rates vary widely across world regions and the disease is more common in countries with well-developed healthcare systems and access to diagnostic tools such as high-resolution ultrasound [46,47]. The rising incidence is due to the variation in geographical regions and environmental exposures. The highest incidence is observed in higher-income countries, such as the Republic of Korea, Canada, Italy, France, Israel, Croatia, Austria and the U.S., as well as some middle- to upper-middle-income countries, such as Turkey, Brazil, Costa Rica and China.

Furthermore, the incidence rates are high in some island nations and territories, including Cyprus, Cabo Verde and French Polynesia [48]. Several risk factors, such as female sex, radiation, dietary iodine content, genetic or hereditary conditions and a history of benign thyroid disease, are associated with an increase in the incidence of thyroid cancer [43]. Metastatic rates for thyroid cancer can vary depending on the histologic subtype of the cancer. PTC generally has a good prognosis and a low mortality rate. The most common site of PTC metastasis is the neck lymph nodes. It can still metastasize to other organs but this is less common. Depending on the stage and other factors, PTC’s regional lymph node metastasis rate can range from 20% to 90%. FTC and MTC have higher metastasis rates and distant metastases can occur. Anaplastic thyroid carcinoma (ATC) is the most aggressive form of thyroid cancer with a high metastatic rate. Surgery is the primary treatment for most cases of thyroid cancer, often followed by radioactive iodine (RAI). Chemotherapy and other targeted therapy drugs, such as sorafenib or lenvatinib, are used for advanced or metastatic thyroid cancer cases that do not respond to standard treatments [32].

The widespread use of imaging modalities, specifically ultrasonography, to evaluate patients has led to a growing number of incidentally discovered thyroid nodules that were previously undetectable [49]. A recent study found that a significant proportion of thyroid cancers (49%) were discovered incidentally in asymptomatic people through histological examination and imaging studies [50]. 

In recent decades, obesity has increased dramatically as it affects all ages, with a worldwide prevalence of 13%; it nearly tripled between 1975 and 2016. The risk of many types of cancer is augmented in morbidly obese individuals with BMI > 40 kg/m^2^ or > 35 kg/m^2^ in the presence of obesity-related comorbidities [7]. Severely obese individuals are more likely to develop thyroid nodules than nonobese individuals [11,51]. Moreover, there is some evidence that adiposity is related to the more aggressive features of thyroid cancer [52,53]. Additionally, type 2 diabetes mellitus (T2DM) is strongly associated with obesity and has been identified as a risk factor for increased TSH levels and thyroid cancer. However, T2DM may be a risk factor for thyroid cancer independent of obesity [11,52]. A 5 kg/m^2^ increase in BMI is correlated with a significant increase in the incidence of PTC [54]. Several studies have suggested a strong association between central adiposity and thyroid cancer [54,55]. Waist circumference is an independent predictor of thyroid cancer [54]. An increase in BMI and waist-to-hip ratio also increases the risk of thyroid cancer. Nevertheless, the connection between obesity and thyroid nodules is unclear due to the different methods used to assess obesity [56]. Despite the above, some studies concluded that there was no significant association between BMI and thyroid cancer [23,57].

## 3. Obesity as a Chronic Inflammatory Condition

It is well established that obesity is a state of chronic low-grade inflammation. During this condition of continuing inflammation, the release of pro-inflammatory factors from tissues increases whereas the release of adipokines decreases, leading to a non-specific immune system activation that is believed to contribute to the development of obesity-related pathologies. The resulting contraposition between adipose-resident immune cells and adipocytes further promotes the immune cell production of multiple pro-inflammatory factors, with subsequent induction of insulin resistance, hyperinsulinemia, hyperglycemia, hyperlipidemia and vascular injury; all of these are associated with oxidative stress, cancer development and progression [7].

Chronic inflammation triggers the activation and transcription of several factors such as nuclear factor-kappa-light-chain-enhancer of activated B cells (NF-κB), signal transducer and activator of transcription (STAT3), and activator protein1 (AP-1) in pre-malignant cells. Obese white tissue increases the production of leptin, which is pro-inflammatory, pro-proliferative, pro-oncogenic and pro-angiogenic. It decreases adiponectin (APN) levels, which are anti-inflammatory, anti-angiogenic and anti-proliferative [58]. Combined with high oxidative stress, inflammatory cytokines are upregulated, enhancing cell proliferation and angiogenesis. The activation of the obese white adipose tissue has multiple roles in cancer, including effects mediated via interleukin IL-1β (IL-1β), which promotes the proliferation and invasion of tumor cells. In addition, the increased levels of circulating free fatty acids observed in obesity may lead to steatosis of the thyroid gland [59].

In rats given a high-fat diet, thyroid tumor cell proliferation was found to be increased via upregulation of cyclin D1 protein levels, phosphorylation of retinoblastoma (Rb) protein, elevated leptin serum levels and increased STAT3 gene expression [7]. Studies also revealed lower APN in patients with thyroid cancer, whereas higher levels of IL-6 and leptin were associated with more advanced papillary thyroid cancer (PTC). APN is shown to be inversely associated with cancer risk in women [7]. 

## 4. Gender and Adipose Tissue

The female sex is a well-known factor associated with the development of thyroid nodules. A study by Lai et al. demonstrated that women with a high body mass index (BMI) are more likely to have thyroid nodules with a high-risk sonographic pattern. The same study revealed a significantly higher risk for thyroid nodules as weight, body fat percentage (BFP), body surface area (BSA) and body mass index (BMI) increased. In addition, statistically significant results were found between the risk of thyroid nodules with a high-suspicion sonographic pattern per 10 kg increase in weight, 5% increase in BSA and 5 kg/m^2^ increase in the BMI [22]. The predominance of thyroid nodules in the female gender in is often attributed to the different patterns of adipose tissue distribution. Song et al. showed that women tend to have larger subcutaneous fat stores than visceral fat, whereas men tend to have more visceral fat for any given waist circumference. Obese individuals, with a higher proportion of subcutaneous fat than visceral fat, are at a lower risk of developing metabolic syndrome [13].

Interestingly, visceral fat is a stronger predictor of insulin resistance among men and women above 50. It is supported that central abdominal obesity carries a higher risk of co-morbid diseases. Lower body adiposity is considered to pose a lower health risk compared with upper body adiposity. There are two theories behind the reasoning that visceral fat is unhealthier. One hypothesis postulates that visceral fat is responsible for the secretion of several adipokines, such as IL-1, IL-6, TNF-α, and leptin. The second hypothesis suggests that the liver is directly exposed to the non-esterified fatty acid released predominately from visceral fat, making it more prone to poor glucose control [60]. 

Adipose tissue serves as an important endocrine organ, and leptin concentration is more reflective of subcutaneous fat, whereas insulin is more indicative of visceral fat. Another important difference is that leptin correlates better with total adipose tissue in women than men. On the other hand, insulin levels are better correlated with adipose tissue in men. Even though women have more fat than men, insulin sensitivity seems less affected by increased body fat than in men. Fat distribution differences among men and women have endocrine, metabolic and health consequences [13,60]. 

**Figure 1:**
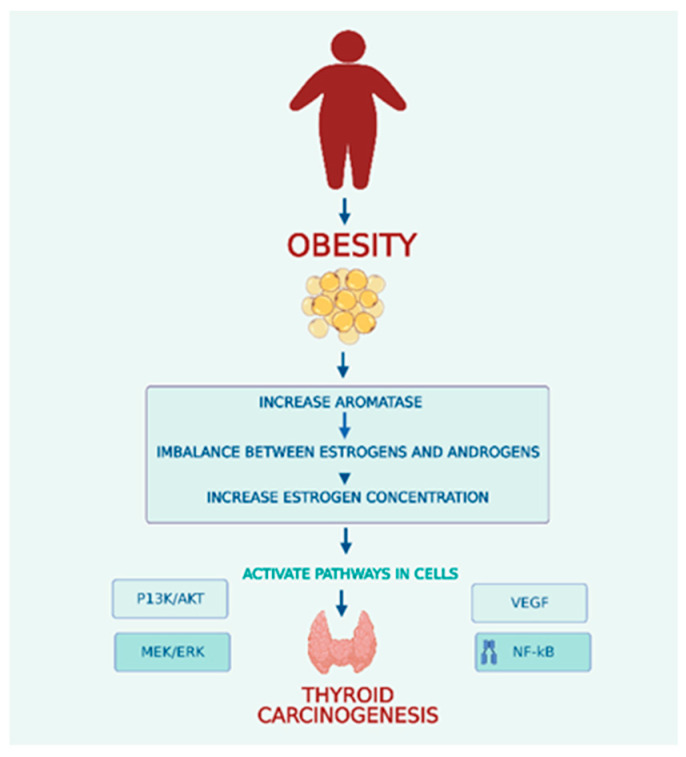
Obesity and Thyroid Carcinogenesis.

Endogenous differences in estrogen levels are considered the major factor for both benign and malignant thyroid nodules (Figure 1). Obesity causes increased aromatase activity in the peripheral adipose, leading to higher levels of circulating estrogens. Hyperactivation of aromatase leads to an imbalance between estrogens and androgens that contributes to carcinogenesis in obese individuals. Two estrogen receptors, ER-α and ER-β, mediate estrogens. ER-α is overexpressed in thyroid cells, whereas ER-β exhibits reduced or absent expression. Several pathways, including PI_3_K/AKT, MEK/ERK, VEGF and NF-k B, are involved in the molecular effects of estrogen in follicular cells [56]. The primary estrogen, 17-beta estradiol (E2), increases cyclin D1 expression by binding to ER-α and reduces the expression of p27 and beta-catenin. E2 can lead to an increased ability for cell proliferation and survival by activation of the Bcl-2 gene [61]. E2 also stimulates reactive oxygen species (ROS) production and promotes the hyperexpression of hypoxia-inducible factor-1α (HIF1-α) [61]. In addition, estrogens can control thyroid tumor development and cancer cell invasiveness through genetic and epigenetic changes and activation of the PI_3_K/AKT and MAPK pathways. Estrogens can also increase angiogenesis by regulating VEGF secretion in thyroid cells. The GPR30 receptor was found in thyroid carcinoma cells and represents another way estrogen stimulates cell growth in cells that lack expression of ERs [61].

In obese postmenopausal women there is an increase in the conversion of endogenous sex steroids. The postmenopausal state is also responsible for a significant imbalance between levels of androgens and estrogens. Postmenopausal women with PTC exhibit an increase in ER-α expression, and these findings suggest a role in promoting cancer aggressiveness. The same increased ER-α expression levels were observed in obese women despite menopause [62].

Adipose tissue is responsible for the conversion or synthesis of endogenous sex steroids through aromatase activity. In obese individuals, the overexpression and hyperactivity of aromatase led to an imbalance between estrogens and androgens, resulting in elevated estrogen levels that contributed to the development of thyroid cancer.

Increases in estrogen concentration activate several cell pathways, including the PI_3_K/AKT, MEK/ERK, VEGF and NF-kB pathways.

PI_3_K/AKT—Phosphatidylinositol-3 kinase signal pathway.

MEK/ERK—Mitogen-activated protein kinase/extracellular signal-regulated kinase.

VEGF—Vascular Endothelial Growth Factor pathway.

NF-Kb—Nuclear Factor kappa B pathway.

## 5. Leptin and the Hypothalamic–Pituitary–Thyroid (HPT) Axis

The stimulation of the HPT axis observed in obesity is mainly due to the centrally acting leptin, which regulates the activity of neurons in the hypothalamus and has both direct and indirect effects on thyrotropin-releasing hormone–stimulating thyroid hormone (TRH-TSH) secretion. Overnutrition caused by hyperleptinemia activates the secretion of TRH and, in turn, promotes the synthesis of TSH and thyroid hormone. Leptin can act directly on the TRHergic neurons within the paraventricular nucleus through the leptin receptor. Indirectly, leptin regulates the mechanism of the hypothalamic neuronal network [63].

In obesity, high leptin levels fail to reduce appetite and increase energy expenditure. Studies in adults showed that leptin-controlled arcuate neurons were unresponsive to signals from adipose tissue and insulin, disrupting energy homeostasis and food intake regulation [63]. Furthermore, leptin is reported to have a significant role in carcinogenesis through stimulating tumor cell growth and invasion by increasing the cellular hypertrophy of thyroid cells and enhancing the expression of thyroglobulin [38]. In vitro studies showed that the stimulating effects of leptin were augmented by the co-administration of TSH [63].

Evidence showed that long-term exposure of the thyroid gland to high levels of TSH due to hyperleptinemia could cause significant thyroid gland hyperplasia and thyroid nodule formation. Furthermore, studies showed that BMI levels, weight circumference (WC), TSH and fasting plasma glucose (FPG) were significantly higher in the thyroid nodule group than in the non-thyroid nodule group. In addition, single-factor logistic regression analysis revealed that thyroid nodule occurrence was positively associated with metabolic syndrome [26]. Leptin receptors in the thyroid gland could induce PTC cell proliferation and inhibit cancer cell apoptosis [7]. The mean TSH levels in obese individuals appeared to be 0.8–2 mU/L higher than in nonobese individuals as a result of the leptin effect on the type II deiodinase in the paraventricular nucleus, which could cause a rise in the incidence of goiter, nodular goiter and papillary thyroid carcinoma development over time [31,58].

## 6. Metabolic Syndrome, Hyperinsulinemia and Insulin-Like Growth Factor-1

Several studies have found an association between metabolic syndrome (MS) components and thyroid nodules [22]. In a quantitative review of thirteen independent observational studies, Zhang et al. reported that metabolic syndrome was related to a higher risk of thyroid nodules [64]. Xu et al., in a large-scale study, found a statistically significant association between thyroid nodules and metabolic disorders. Overweight, central obesity, high blood glucose, hypercholesterolemia and fatty liver disease were all significantly associated with the prevalence of multiple thyroid nodules [38]. A positive relationship was found between all components of metabolic syndrome and nodular goiter; however, the most significant association was reported between insulin resistance, thyroid nodules and PTC [58]. In the study by Moon et al., the prevalence of metabolic syndrome was higher in the patient group with nodules; both higher BMI and waist circumference were independently associated with thyroid nodules [31]. IGF-1 promotes the progression and mitosis of many types of cells together with signal transduction through the insulin-like growth factor 1 receptor (IGF1-R), leading to abnormal hyperplasia, differentiation and apoptosis of thyrocytes. This pathway could be a TSH-independent mechanism explaining the association between thyroid nodule formation and metabolic syndrome [65].

According to Chen et al., insulin resistance caused by obesity could result in steatosis and infiltration of the thyroid gland, subsequently leading to changes in the gland’s morphology and function [59]. Insulin induces tissue hyperplasia by stimulating cell division and prolongs cell survival by exerting anti-apoptotic effects. In addition, thyroid cancer tissues had higher insulin receptor levels [58].

The insulin receptor (IR) and IGF-R are structurally homologous. When insulin binds to its receptor, the PI_3_K pathway is activated, leading to glucose uptake, whereas the MAPK pathway is responsible for cell and tissue proliferation and gene expression. The MAPK pathway has a major role in insulin resistance and chronic hyperinsulinemia. The activation of insulin growth factor receptors is believed to play an important role in various types of cancer, with an increased incidence of metabolic syndrome. In a state of insulin resistance, the amount of IGF-binding protein is reduced, resulting in high levels of circulating IGF [58]. 

IGF-R is present at a higher density on the membranes of cold nodules than hyperactive and normoactive ones. Additionally, higher levels of insulin receptors were detected in differentiated and undifferentiated thyroid cancer tissues. Interestingly, the addition of metformin, an insulin sensitizer, inhibits tumor cell proliferation, which is associated with the activation of the adenosine monophosphate kinase pathway in cells and subsequent inhibition of the mammalian target rapamycin (mTOR) pathway. Metformin is thought to be protective against thyroid cancer development [56,58]. Liu et al. reported that insulin resistance was closely related to the distribution, density and structure of the thyroid blood vessels, which might promote the development of thyroid nodules [26].

Shin et al. showed that the tumorigenic effect of metabolic syndrome appears to be mediated through changes in cytokine levels due to low expression of tumor suppressor genes and an increase in the levels of oncogene expression due to inflammation and ROS production. A higher prevalence of large nodules in individuals with metabolic syndrome and hyperglycemia indicated by glycated hemoglobin (HbA1C) levels demonstrates a significant relationship with thyroid nodules in women [65].

## 7. Adipose Tissue and TSH Levels

Several epidemiological studies have noted a positive correlation between elevated TSH levels and BMI. In patients with metabolic syndrome and subclinical hypothyroidism, serum TSH levels were correlated with the severity of obesity. TSH can promote the differentiation of pre-adipocytes into mature adipocytes. TSH was found to increase the accumulation of triglycerides by thyroid-stimulating hormone receptors (TSHR) via activation of the AMPK/PPARγ pathway. Thyrotropin and TSHR were thought to be limited only to thyrocytes. Nevertheless, the expression of TSHR in extrathyroidal tissues, including the liver and adipose tissue, was also demonstrated [66].

Patients with MS have a higher prevalence of nodular thyroid disease, probably due to inflammatory levels and elevated TSH [67]. Glucose tolerance was demonstrated to have the most significant contribution to the development of nodules. Activation of the TSH receptor and the production of intracellular cyclic AMP (cAMP) leads to the activation of protein kinase A (PKA), which phosphorylates the nuclear factor cAMP response element binding protein (CREB), which, in turn, activates the targeted genes leading to thyroid cell proliferation. Elevated TSH can increase thyroid cell proliferation through p70S6K-mediated effects on the localization of p27 [68]. Insulin activates (IGF)-1 receptors that can increase the sensitivity of TSH, promoting thyroid cell proliferation. TSH possesses well-known mitogenic properties in the presence of insulin in cell cultures [67].

## 8. Oxidative Stress and Diet

The low-grade chronic inflammation associated with obesity is responsible for many pathological conditions, including hyperglycemia, hyperlipidemia, hyperinsulinemia, insulin resistance and vascular damage, that are all related to oxidative stress, which leads to carcinogenesis. Excess harmful free radicals and reactive molecules characterize oxidative stress. The exact function of oxidative stress in thyroid cancer remains uncertain; nonetheless, there are indications that a plausible link exists between decreased antioxidant activity and augmented generation of reactive oxygen species (ROS), which may contribute to the development of thyroid tumors. ROS activation of the MAPK and PI_3_K/AKT signaling pathways could lead to tumorigenesis [56]. 

Diet has a significant role in the pathophysiology of chronic inflammation; the dietary inflammatory index (DII) is a measure that explores the correlation between diet and biomarkers of inflammation. Two recent studies have linked high DII scores to thyroid cancer and other inflammatory conditions [56]. According to a study by Luo et al., intentional weight loss among postmenopausal women in the Women’s Health Initiative (WHI) study was associated with a decreased risk of developing obesity-related cancer. The WHI study consisted of 58,667 postmenopausal women aged 50–79, whose body weight and waist circumference were measured at baseline and year 3. During the average 12-year follow-up period, 6033 cases of obesity-related cancers were reported [69]. A meta-analysis of 24 million cohorts that was conducted to evaluate the association between obesity and weight change and the risk of developing thyroid cancer demonstrated that obesity and weight gain are significant risk factors for thyroid cancer. Specifically, obesity increases the risk of thyroid cancer in women and, conversely, maintaining a healthy weight was associated with a reduced risk of thyroid cancer in both men and women [70]. 

## 9. Conclusions and Future Perspectives

**Figure 2:**
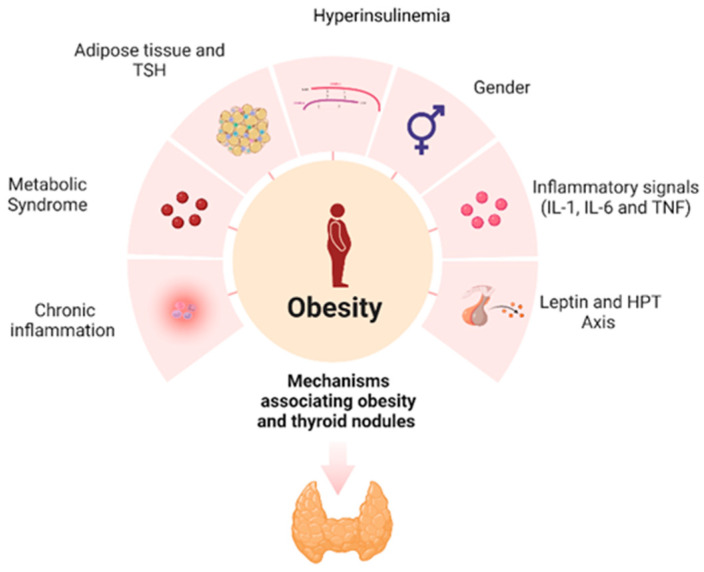
Mechanisms associating obesity and thyroid nodules.

The pathophysiology of thyroid nodules and thyroid cancer is complex, with various parameters and biological pathways involved. The correlation between obesity and thyroid cancer is established and can be explained, at least in part, through a plethora of molecular mechanisms. The main mechanisms linking obesity with the development of thyroid nodules are shown in Figure 2. Obesity alone, as a state of chronic inflammation, is a key factor in the pathophysiology of thyroid nodules. Obesity, through increased leptin secretion, insulin resistance and activation of the HPT axis, contributes to the genesis and development of thyroid nodules. Further investigation is needed to elucidate the mechanisms involved in the multifactorial link between thyroid nodules, thyroid cancer, obesity and metabolic syndrome. 

Obesity, a state of chronic, low-grade inflammation, causes an immune response that promotes carcinogenesis. Leptin plays a vital role in the development and progression of thyroid nodules; estrogen is also involved in thyroid nodule formation. Obesity results in increased levels of circulating estrogens due to increased aromatase activity. Metabolic syndrome stimulates proliferation and angiogenesis in the thyroid gland through hyperinsulinemia, hyperglycemia and dyslipidemia. Thyroid-stimulating hormone (TSH) can turn pre-adipocytes into mature adipocytes and exerts mitogenic effects.

Potential future perspectives in better understanding the association between obesity and thyroid nodules will help clinicians to form new risk assessment and screening strategies. Obese individuals may be considered at higher risk for developing thyroid nodules, prompting healthcare professionals to include thyroid examinations in routine check-ups or screening programs that lead to early detection and timely intervention. By understanding the association, healthcare professionals can stratify the risk for developing thyroid nodules in obese individuals. This will help identify high-risk individuals and focus on frequent monitoring and targeted interventions to prevent the development or progression of nodules. There are also implications on the treatment approaches by healthcare professionals, who may consider more aggressive management strategies such as earlier consideration of fine-needle aspiration or surgery depending on the characteristics of the nodules. Better understanding and establishing the links between obesity, thyroid nodules and thyroid cancer can help educate patients about the potential risks and motivate them to make positive lifestyle changes. This knowledge can empower patients to adopt healthy habits, follow a balanced diet, increase physical activity, lose weight, modify their lifestyle and actively participate in their health. Understanding the link between obesity and thyroid nodules can spur further research into targeted interventions. This integrated approach can improve overall health outcomes by addressing multiple risk factors and comorbidities. 

Future directions for research include large-scale studies to assess the impact of weight loss on thyroid cancer in obese individuals. Although epidemiological studies suggest a link between obesity and thyroid cancer, further research is needed to fully understand the mechanisms underlying this association and develop effective prevention and treatment strategies based on this knowledge. Additionally, randomized controlled trials are needed to assess the effects of targeting obesity and its potential mediators such as inflammation, insulin resistance, IGF-1 and low adiponectin on thyroid cancer prevention. However, there are challenges, such as disentangling the effects of obesity from other factors, the heterogeneity of thyroid cancers and a lack of consensus on specific mechanisms. 

Ongoing research can lead to a better understanding of disease and the development of more effective prevention and treatment strategies. Nonetheless, given that obesity is a risk factor that can be modified, it is an important target for public health initiatives to reduce the incidence of thyroid cancer and the formation of thyroid nodules. Encouraging healthy weight management and weight loss through public health initiatives can help lower the risk of developing thyroid cancer.

## Data Availability

Not applicable.
